# The Energy Coding of a Structural Neural Network Based on the Hodgkin–Huxley Model

**DOI:** 10.3389/fnins.2018.00122

**Published:** 2018-03-01

**Authors:** Zhenyu Zhu, Rubin Wang, Fengyun Zhu

**Affiliations:** Institute for Cognitive Neurodynamics, East China University of Science and Technology, Shanghai, China

**Keywords:** neural energy coding, energy distribution, synchronous oscillation, negative energy, power consumption curve

## Abstract

Based on the Hodgkin-Huxley model, the present study established a fully connected structural neural network to simulate the neural activity and energy consumption of the network by neural energy coding theory. The numerical simulation result showed that the periodicity of the network energy distribution was positively correlated to the number of neurons and coupling strength, but negatively correlated to signal transmitting delay. Moreover, a relationship was established between the energy distribution feature and the synchronous oscillation of the neural network, which showed that when the proportion of negative energy in power consumption curve was high, the synchronous oscillation of the neural network was apparent. In addition, comparison with the simulation result of structural neural network based on the Wang-Zhang biophysical model of neurons showed that both models were essentially consistent.

## Introduction

Currently, in neuroscience, several conventional encoding theories and decoding technologies are followed (Amari and Nakahara, 2005; Purushothaman and Bradley, 2005; Natarajan et al., 2008; Gazzaniga et al., 2009; Jacobs et al., 2009). However, none of these theories and technologies are well-established globally for analyzing the brain activity (Laughlin and Sejnowski, 2003; Abbott, 2008; Wang and Zhu, 2016). Our study shows that energy acting as a carrier throughout all the brain activities provides a novel direction for understanding the cognitive neuroscience and neural information processing. Both suprathreshold and subthreshold neural activities are accompanied by energy consumption; however, the relationship between the pattern of brain energy consumption and perceptual cognition is not yet clarified. From the perspective of global brain activity, the neural activities which response to environmentally driven demands account for less than 5% of the brain's energy budget, leaving the majority devoted to intrinsic neural signaling (Raichle and Mintun, 2006; Sokoloff, 2008; Zhang and Raichle, 2010). As for the local neuronal firing, less than 40% of signaling-related energy consumption is for the housekeeping mechanism and the maintenance of resting potentials of both neuronal and glial cells, while the other is for the action potentials and postsynaptic potentials (Attwell and Laughlin, 2001; Howarth et al., 2012; Yu et al., 2017). This highly mismatched ratio of energy consumption implies that an important mechanism associated with cognitive neural activity underlies the energy consumption of the brain, but is yet to be elucidated.

The neural activity and operations of the brain obey the principle of minimizing the energy consumption and maximizing the signal transmission efficiency (Laughlin and Sejnowski, 2003). And the collaboration of sodium channels and potassium channels contributes substantially to energy-efficient metabolism by minimizing the overlap of their respective ion fluxes (Alle et al., 2009). Owing to these operations, a novel neural coding theory, termed as neural energy coding, has been proposed in the field of neuroinformatics in recent years (Wang et al., 2006, 2008, 2016, 2017; Wang and Wang, 2014; Wang R. et al., 2014; Wang Z. et al., 2014; Yan et al., 2016). This coding theory states that membrane potential of the neuron uniquely corresponds to its consumed neural energy, which enables us to transform the complex and highly nonlinear spiking pattern of membrane potentials into the distribution pattern of energy to deal with neural activity (Abbott, 2008; Wang et al., 2008; Wang and Wang, 2014; Wang R. et al., 2014; Wang Z. et al., 2014). According to this theory, the neural energy coding can serve as the foundation for global neural coding of brain function (Wang et al., 2006; Wang and Zhu, 2016; Zheng et al., 2016) because of the following reasons: (1) Energy can be used for analyzing and describing the neuroscientific experiments at various levels such that the computational results are no longer mutually unavailable, contradictory, and irrelevant (Wang and Zhu, 2016). Thus, the neural information can be expressed as energy at the level of molecule, neuron, network, cognition, behavior, and their combination such that it can unify the neural models among various levels. (2) The neural energy can be combined with spiking pattern of membrane potentials to resolve the neural information (Wang et al., 2006, 2008, 2016, 2017; Wang and Wang, 2014; Wang R. et al., 2014; Wang Z. et al., 2014; Kozma, 2016; Yan et al., 2016; Zheng et al., 2016). (3) Neural energy can describe the interaction of large-scale neurons referring to the interaction of multiple brain regions that cannot be achieved by any conventional coding theory (Wang et al., 2009; Vuksanović and Hövel, 2016; Zhang et al., 2016; Déli et al., 2017; Peters et al., 2017). (4) Currently, a simultaneous recording from multiple brain regions in traumatic brain injury experiments is challenging. Although EEG and MEG can sample the neuronal activity from various brain regions, it is difficult to estimate the cortical interactions based on these extracranial signals. The main obstacle is the lack of a theoretical tool to effectively analyze the interaction between cortices in a high dimensional space (Hipp et al., 2011) and transform the scalp EEG into cortical potential. Nevertheless, neural energy resolves this issue (Wang and Wang, 2017). (5) Whether the neural model is based on a single neuron, neural populations, networks or behaviors and is linear or nonlinear, their dynamic response can describe the pattern of neural coding by energy superposition owing to the scalar property of energy (Wang et al., 2006, 2008, 2016, 2017; Wang and Wang, 2014; Wang R. et al., 2014; Wang Z. et al., 2014; Yan et al., 2016; Zheng et al., 2016). Thus, the global information of functional brain activity can be acquired, which is not achieved by the other traditional coding theories. (6) Despite the ever-changing pattern of network oscillation, the uniquely corresponding relationship between network oscillations and energy oscillations greatly facilitates the modeling and numerical analysis of a large scale of neural networks with large dimensions and strong nonlinearity. This phenomenon is effectuated by neural energy coding such that the complex neuroinformatics becomes easy to handle without losing information.

This coding theory has made several research findings possible (Wang et al., 2006, 2008, 2016, 2017; Wang and Wang, 2014; Wang R. et al., 2014; Wang Z. et al., 2014; Du et al., 2016; Wang and Zhu, 2016; Yan et al., 2016; Zheng et al., 2016). Previously, Wang and Zhang proposed a novel biophysical model of neurons, which provides the membrane potential function of a single neuron and the corresponding energy function with their calculation method (Wang et al., 2006). The comparison with the Hodgkin–Huxley (H–H) model reveal that both models are essentially consistent (Wang R. et al., 2017). Subsequently, we investigated the energy coding of a structural neural network and captured its energy distribution feature under different parameters (Wang and Wang, 2014; Wang R. et al., 2014; Wang Z. et al., 2014). Recently, we applied this novel neural coding theory to mental exploration by regarding the spatial distribution of the power of place cells in hippocampus as a type of neural energy field. The result showed that a nearly optimal exploration path can be found only by about ten times of mental exploration. Compared to the conventional studies on mental exploration, this method of neural energy field gradients greatly improves the efficiency of mental exploration (Wang et al., 2016).

In our previous study, the structural network model was based on the Wang–Zhang biophysical model of neurons (Wang et al., 2006). Herein, we explore the energy consumption property of structural neural networks based on the classical H–H model in order to lay the foundation of neural energy in the field of computational neuroscience. Then, according to the dynamic feature that neurons firstly absorb energy followed by consumption while firing the action potentials, we propose a vital index defined as the ratio of cumulatively stored energy to cumulatively consumed energy in order to quantitatively analyze the synchronization of neural activity. Moreover, the comparison with the previous studies (Wang et al., 2006; Wang and Wang, 2014) lay a theoretical foundation for the future investigation of the global brain function neural model and large scale global neural coding.

## Computational model

### Energy consumption of the H–H neuron

**Figure 1:**
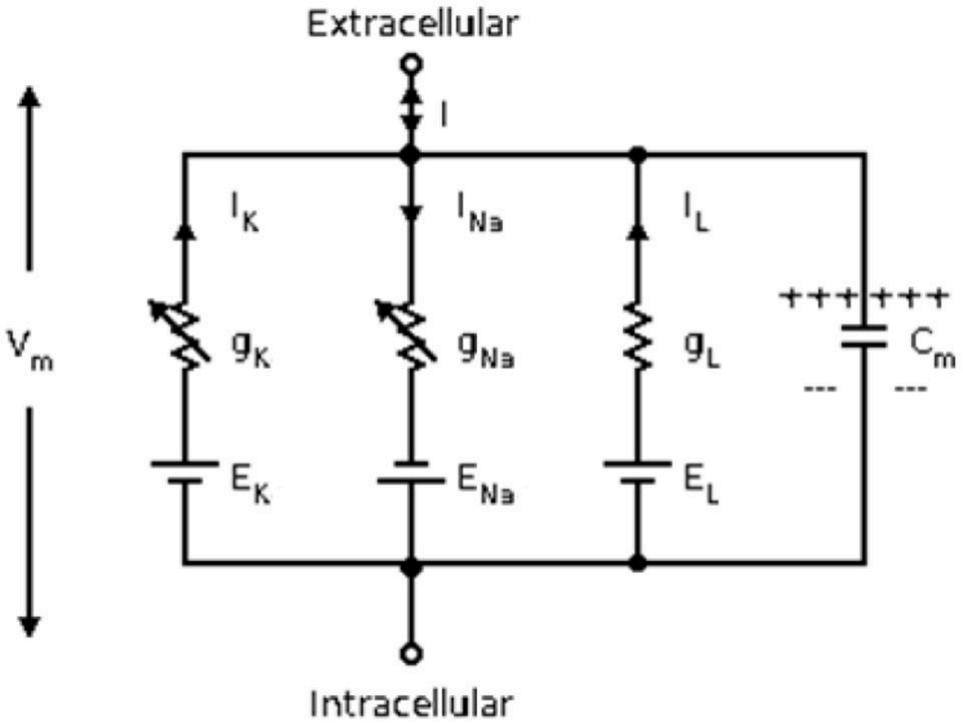
The equivalent circuit diagram of the Hodgkin–Huxley model.

Herein, the classical Hodgkin–Huxley model (Hodgkin and Huxley, 1990) is used for calculating the energy consumption of neurons. The equivalent circuit diagram of Hodgkin–Huxley model is shown in Figure 1. The differential equation is described as follows:

(1) $$\begin{array}{l} C_{m}\frac{dV_{m}}{dt}=g_{l}\left(E_{l}-V_{m}\right)+g_{Na}m^{3}h\left(E_{Na}-V_{m}\right)\\ +\;g_{K}n^{4}\left(E_{K}-V_{m}\right)+I \end{array}$$

Where *C*_*m*_ is the membrane capacitance, *V*_*m*_ is the membrane potential, *E*_*Na*_ and *E*_*K*_ are the Nernst potentials of sodium and potassium ions, respectively, and *E*_*l*_ is the potential at the time when the leakage current is zero. In addition, *g*_*l*_ is the leakage conductance, *g*_*Na*_ and *g*_*K*_ are the maximum conductance of sodium and potassium ion channels, respectively. The two variable conductances are described by the following set of nonlinear differential equations:

(2) $$\begin{array}{l} \{\begin{array}{c} \frac{dn}{dt}=\alpha_{n}(1-n)-\beta_{n}n\\ \frac{dm}{dt}=\alpha_{m}(1-m)-\beta_{m}m\\ \frac{dh}{dt}=\alpha_{h}(1-h)-\beta_{h}h \end{array} \end{array}$$

where:

$$\begin{array}{l} \alpha_{n}=\frac{0.01\left(10+V_{m}-V_{r}\right)}{\exp\left[\left(\left(10+V_{m}-V_{r}\right)/10\right)-1\right]};\\ \beta_{n}=0.125\exp\left(\frac{V_{m}-V_{r}}{80}\right);\\ \alpha_{m}=\frac{0.1\left(25+V_{m}-V_{r}\right)}{\exp\left[\left(\left(25+V_{m}-V_{r}\right)/10\right)-1\right]};\\ \beta_{m}=4\exp\left(\frac{V_{m}-V_{r}}{18}\right)\\ \alpha_{h}=0.07\exp\left(\frac{V_{m}-V_{r}}{20}\right);\\ \beta_{h}=\frac{1}{\exp\left[\left(30+V_{m}-V_{r}\right)/10+1\right]} \end{array}$$

V_*r*_ is resting potential.

The procedure involves the following steps for the neuron firing action potentials: (1) The postsynaptic neurons are stimulated by presynaptic neurons, which increases the permeability of the cell membrane to sodium ions. Then, the sodium ions begin to flow inward, while the membrane potential approaches the threshold for the preparation of depolarization (subthreshold activity); (2) The permeability of the cell membrane to sodium ions increases further, and the sodium ions flow inward largely, while the membrane potential increases rapidly (suprathreshold activity); (3) The permeability of the cell membrane to sodium ions are weakened, while the permeability to potassium ions increases such that the potassium ions flow outward. Then, the membrane potential begins to decrease after reaching the peak to perform repolarization; (4) The permeability of the cell membrane to potassium ions increases further such that they flow outward until hyperpolarized; (5) The permeability of the membrane to potassium decreases, and membrane potential increases to the level of resting.

The flow of ions include active and passive transport across the membrane. Active transport is that the sodium-potassium pump consumes ATP to carry in potassium ions and carry out sodium ions of the membrane, while passive transport is that ions flow with the driving force of the gradient differences of concentration and potential (Zheng et al., 2014). Ion diffusion through the ion channels doesn't consume biological energy under the effect of concentration gradient. And the flow of ions under the effect of potential gradient is actually caused by the work of electric field force rather than the consumption of ATP. Thus, only the energy consumed by active transport should be regarded as the biological energy consumed by the neuron.

Since the energy consumed by active transport can't be directly calculated, we firstly consider the energy of the whole electric circuit of the Hodgkin–Huxley neuron. The electric energy is accumulated in membrane capacitor and equivalent batteries generated by Nernst potentials of ions at a particular moment (Moujahid et al., 2011), which can be expressed as:

$$\begin{array}{l} E_{all}\left(t\right)=\frac{1}{2}C_{m}V_{m}^{2}+H_{Na}+H_{K}+H_{l} \end{array}$$

Due to the difficulty of calculating the last three terms, we get the first order differential of *E*_*all*_(*t*) to focus on the electric power:

$$\begin{array}{l} P_{all}\left(t\right)=\frac{dE_{all}}{dt}=C_{m}\frac{dV}{dt}V_{m}+P_{Na}+P_{K}+P_{l} \end{array}$$

where the last three terms are the power of equivalent batteries represented by Nernst potentials of ions.

While

$$\begin{array}{l} C_{m}\frac{dV}{dt}=I-i_{Na}-i_{K}-i_{l}, \end{array}$$

thus,

$$\begin{array}{l} P_{all}\left(t\right)=IV_{m}+P_{Na}+P_{K}+P_{l}-V_{m}\left(i_{Na}+i_{K}+i_{l}\right) \end{array}$$

in which, *IV*_*m*_ is the power of external stimuli, (*P*_*Na*_ + *P*_*K*_ + *P*_*l*_) is the power of the voltage sources represented by the Nernst potentials, whereas *V*_*m*_(*i*_*Na*_ + *i*_*K*_ + *i*_*l*_) is the power consumed by the driving force of the membrane potential gradient (electric field force), which should be regarded as the power of passive transport. However, in the course of the firing action potentials by neurons, if the energy consumed by the changes in the permeability of the cell membrane is not considered, then the involved energy includes the energy provided by surrounding environments, the energy in the potential differences between the internal and external cell membrane, as well as the biological energy (ATP) consumed by the ions pump. During the transformation of subthreshold neurons into functional neurons, the sum of these three types of energy is dynamically equivalent to the total energy in the circuit system of H–H model. The former two types correspond to *IV*_*m*_ and *V*_*m*_(*i*_*Na*_ + *i*_*K*_ + *i*_*l*_) in the circuit, respectively. Then, the power (*P*_*Na*_ + *P*_*K*_ + *P*_*l*_) of the voltage source represented by the Nernst potential is nearly equal to the biological power of the sodium-potassium pump which is the power consumed by the neuron. In this process, the sodium-potassium pump continually transports ions, thereby directly consuming the biological energy which means that 1 ATP can pump out three sodium ions and pump in two potassium ions (Attwell and Laughlin, 2001). This also confirms that the existence of the ion pump helps to maintain the Nernst potential (Laughlin et al., 1998) by continuously transporting ions. As the sodium ions flow inward and the potassium ions flow outward, the membrane potential goes up above zero, and the sodium-potassium pump can pump out sodium ions with the help of potential difference but pump into potassium ions against potential difference. It can be regarded as that the voltage source represented by the Nernst potential of sodium ions is storing energy, while the counterpart of potassium ions is consuming energy. Consequently, *P*_*Na*_ is negative but *P*_*K*_ is positive. The situation about *P*_*l*_ is the same as*P*_*K*_. Therefore, we can calculate the power consumed by the ion pump through the power of voltage source represented by the Nernst potential as below:

(3) $$\begin{array}{l} P=P_{Na}+P_{K}+P_{l}=|i_{K}E_{K}|+|i_{l}E_{l}|-|i_{Na}E_{Na}| \end{array}$$

**Figure 2:**
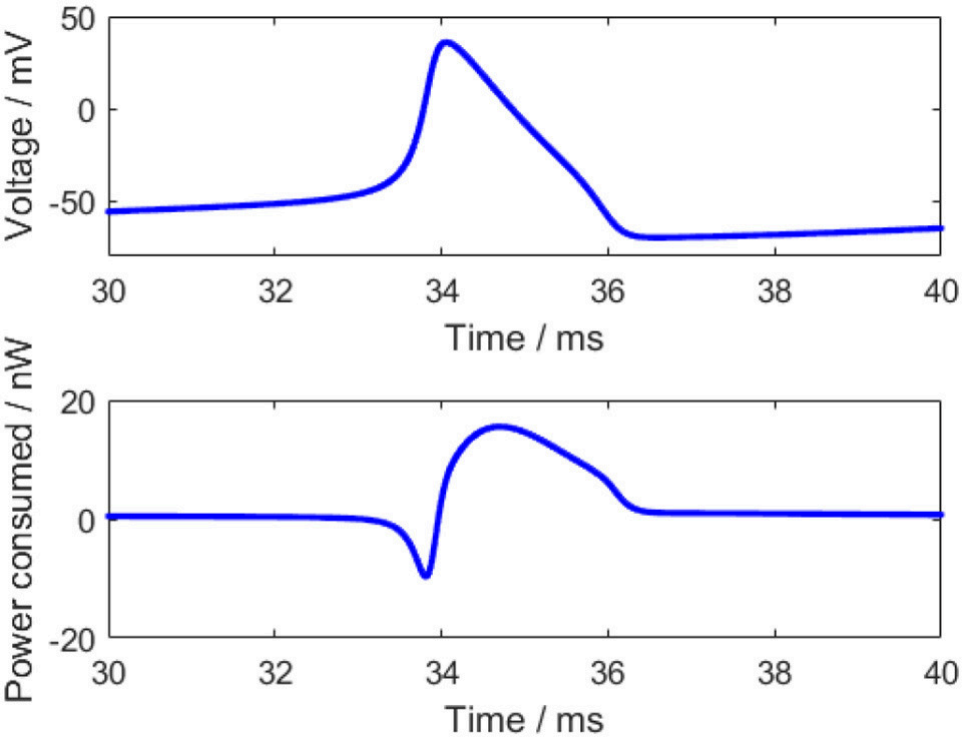
The action potential and corresponding power consumption curve.

For an action potential, the neuronal energy consumption can be calculated by the above equation (Figure 2). All parameter values in the calculation refer to the experimental values (Hodgkin and Huxley, 1990) and are shown in Table 1.

**Table 1:** The parameter values in the calculation.

**Ion**	**Nernst potential/mV**	**Maximum conductance/mS/cm^2^**
*Na*	55	120
*K*	−72	36
*L*	−50	0.3
Membrane capacitance/μF/cm^2^		1
Resting potential/mV		−60

The power consumption curve of the neuron can be obtained as shown in Figure 2 according to Equation (3). At the start of the action potential, the neuron absorbs some energy from ATP hydrolysis. Meanwhile, the sodium-potassium pump works weakly due to a few sodium ions and a great many potassium ions inside the membrane, thereby consuming merely a small amount of energy. Thus, the energy absorbed by the neuron is more than the energy consumed by the sodium-potassium pump in the initial stage of the action potential, which is reflected by the negative region in the power consumption curve before the peak of the membrane potential. As the membrane potential goes up, there accumulates a large number of sodium ions gradually inside the membrane and some potassium ions flow outward, which increases the work demand of the sodium-potassium pump that can pump out the sodium ions and pump in the potassium ions to maintain the Nernst potential (Laughlin et al., 1998). Hence, the power consumption of the neuron begins to change toward positive and the energy consumption is greatly increased when the membrane potential reaches the peak, and the peak of the energy consumption lags behind the peak of the membrane potential (~0.4 ms), which is consistent with the previous results (Wang et al., 2006; Wang R. et al., 2014). Importantly, the positive and negative regions of this power consumption curve have profound neurobiological significance that they correspond to the experimental result, which states that the blood flow rises by about 31% while the accompanying oxygen consumption increases by only 6% in the stimulation-induced neural activity (Lin et al., 2010; Tozzi and Peters, 2017).

### Structural neural network model

**Figure 3:**
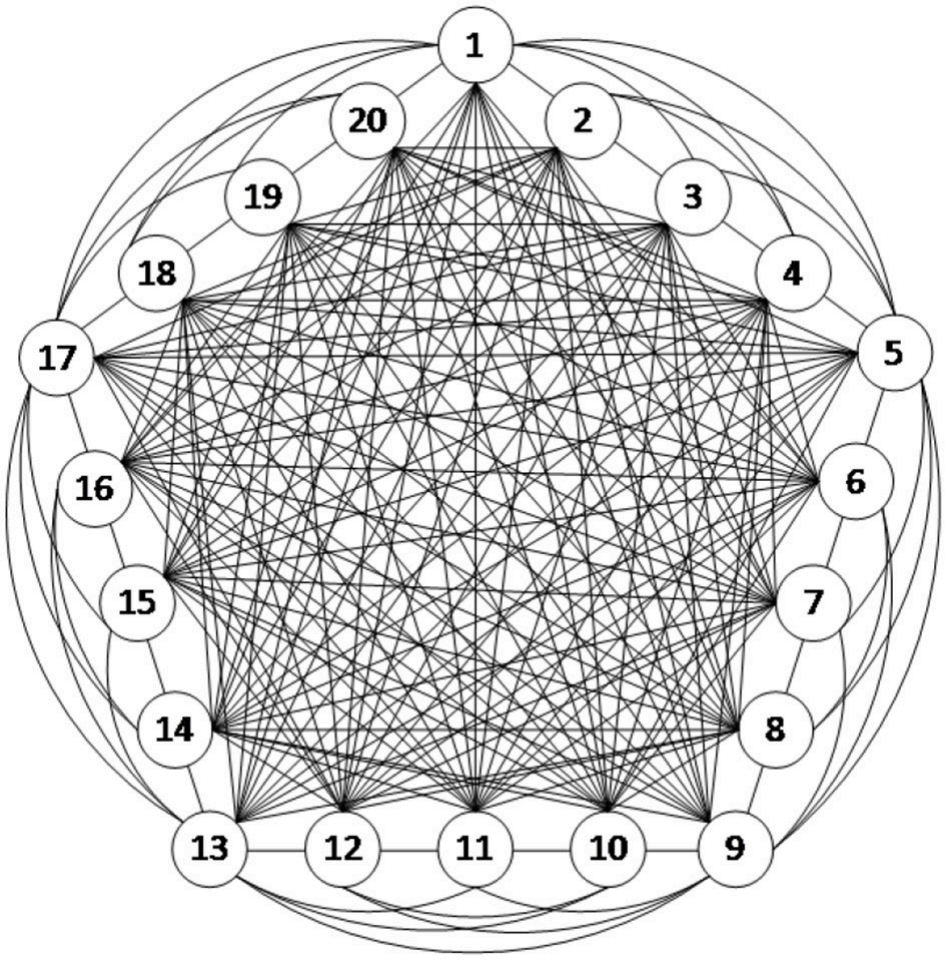
The schematic of a fully connected structural neural network.

In this study, the connection structure of the neural network is illustrated in Figure 3. The dynamic characteristics of each neuron are represented by the H–H model as described above, and thus, the network structure is strictly defined neurobiologically. Figure 3 is an example of a fully connected neural network consisting of 20 excitatory neurons. Since the scope of this article focused on understanding the energy coding pattern of the neural network under different parameters, the neural network connection is simplified to some degree. The neurons are connected with bidirectional and asymmetrical coupling strengths. According to the principle of synaptic plasticity, the statistical data from the experiments demonstrate that the synaptic coupling strength between the neurons is uniformly distributed (Rubinov et al., 2011).

The coupling strength matrix:

$$\begin{array}{l} W=\left[\begin{array}{cccc} w_{1,1} & w_{1,2} & \ldots & w_{1,n}\\ w_{2,1} & \ddots & \; & w_{2,n}\\ \vdots & \; & \ddots & \vdots \\ w_{n,1} & w_{n,2} & \ldots & w_{n,n} \end{array}\right], \end{array}$$

*w*_*i,j*_ is coupling strength when the *i*th neuron is coupled to the *j*th neuron, and *n* is the number of neurons in the network.

The network operates as follows:

(4) $$\begin{array}{l} I_{in}\left(t\right)=W\times Q{\left(t-\tau \right)}^{\prime }\\ I\left(t\right)=I_{in}\left(t\right)+I_{ext}\left(t\right) \end{array}$$

where *I*(*t*) is the sum of stimulated currents to the neuron at time*t*, *I*_*in*_(*t*) is the interaction between neurons, and *I*_*ext*_(*t*) is the external stimulated current to the neuron; *Q*(*t* − τ) = [*Q*_1_(*t* − τ), *Q*_2_(*t* − τ), …, *Q*_*j*_(*t* − τ), …*Q*_*n*_(*t* − τ)], indicating the firing state of each neuron at time *t* − τ, which assigns the value of 0 at resting and 1 at firing. τ indicates the interval from the presynaptic neuron firing a spike to the postsynaptic neuron receiving stimulus, which is the signal transmitting delay. In this study, its value is subjected to a uniform distribution.

### Synchronization index

In order to quantitatively estimate the synchronization of the network activity, the traditional synchronization index of the mean-max correlation coefficient (MCC) and the novel negative energy ratio were used in this article.

The MCC is defined as follow:

$$\begin{array}{l} \rho_{mean}=\frac{\overset{N}{\underset{i=1}{\sum }}\text{max}\left(C_{i,1},C_{i,2},\ldots C_{i,j},\ldots ,C_{i,n}\right)}{N}\;\left(i\neq j\right) \end{array}$$

Where *C*_*i,j*_ is the Pearson correlation coefficient between the membrane potentials of the *i*th and *j*th neurons. If the Pearson's correlation coefficient between any two neurons is closer to 1, the synchronization between these two neurons is stronger. Previous studies have found that if the network reaches a steady state under the transient stimulus, two or more synchronous oscillation groups might occur (Wang and Wang, 2014). It can be seen that the approaching to 1 of MCC denotes that the synchronization within the oscillation group is salient, and the network coexists multiple synchronous oscillation groups. On the other hand, the approaching to 0 of MCC denotes that the synchronization within the oscillation group is weak and only a subset of neurons are in a synchronized state.

The negative energy ratio is defined as the ratio of negative energy to the sum of negative and positive energy consumed by the network during the period from moment 0 to *t*; i.e.,

$$\begin{array}{l} \alpha \left(t\right)=\frac{E_{negtive}}{E_{positive}+E_{negtive}}\times 100\%\\ E_{negtive}=\overset{n}{\underset{i=1}{\sum }}\int_{o}^{t}P_{i}\left(t\right)\cdot \text{ sgn}\left(-P_{i}\left(t\right)\right)dt\\ E_{positive}=\overset{n}{\underset{i=1}{\sum }}\int_{o}^{t}P_{i}\left(t\right)\cdot \text{ sgn}\left(P_{i}\left(t\right)\right)dt \end{array}$$

Where *P*_*i*_(*t*) is the power consumed by the *i*th neuron at time *t*, and the integration of *P*_*i*_(*t*) in [0,*t*] represents the energy consumption during this period. sgn(*x*) is the sign function which is defined as $\text{sgn}(x)=\{\begin{array}{ll} 1, & x>0\\ 0, & x\leq 0 \end{array}.E_{negtive}$ and *E*_*positive*_ represent the negative and positive energies, respectively, consumed by the network in [0,*t*].

## Results and discussion

### Energy consumption property during oscillation in various parameters

#### The energy consumption property of the neural network with different sizes under continuous stimulus

**Figure 4:**
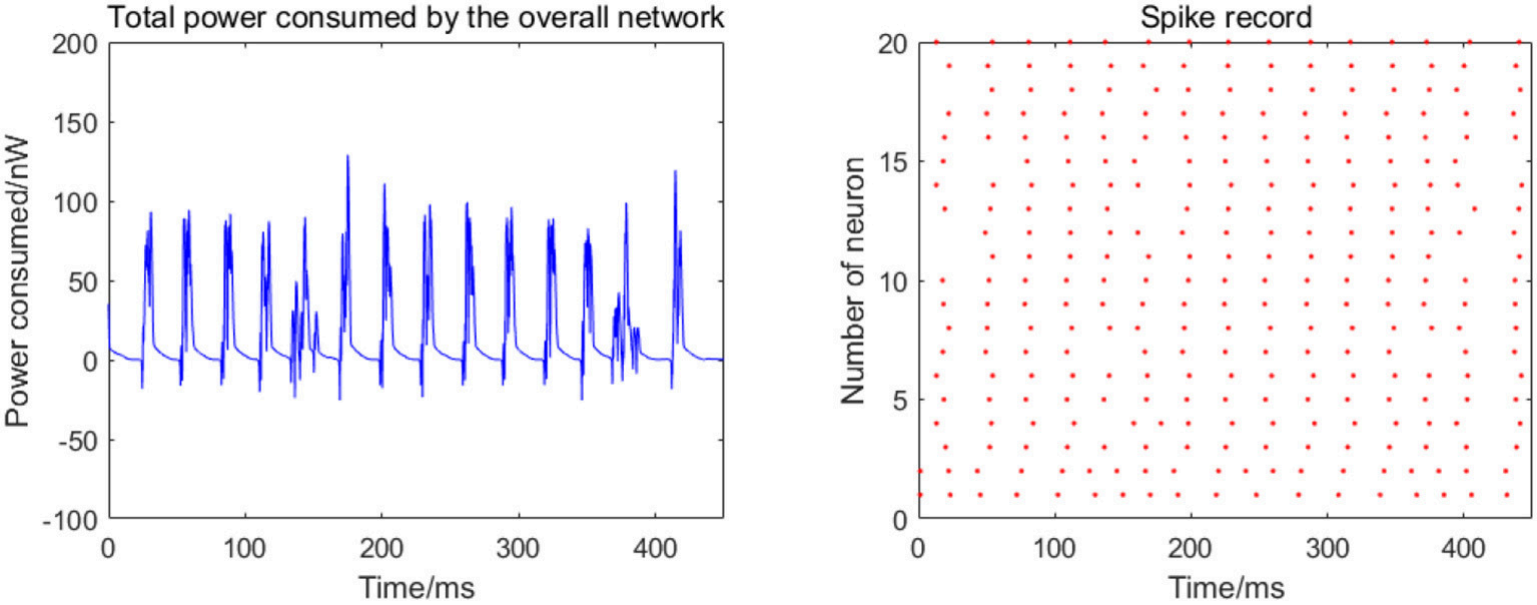
Total power consumed by the overall network of 20 neurons and the spike record under continuous stimulus. The coupling strength is uniformly distributed in [0, 0.5]; the signal transmitting delay is uniformly distributed in [0.3 ms, 1.8 ms].

Given the fully connected neural network consisting of 20 neurons, the total energy consumed by the overall network and the spike record under continuous stimulus is shown in Figure 4.

The 20 neurons are numbered from 1 to 20, and the coupling strength between the neurons is uniformly distributed in [0, 0.5], and the signal transmitting delay is uniformly distributed in [0.3 ms, 1.8 ms]. The 1st and 2nd neurons are continuously stimulated with the intensity of 10 μA/cm^2^ from *t* = 0–450 ms. The left panel in Figure 4 shows the total energy consumed by the overall network during the simulation, and the right records the spike within the first 250 ms (the red dot at the coordinate (*t*,*i*) represents the *i*th firing a spike at time*t*). The more streak-like the record, the stronger the synchronization of the network.

According to the left panel in Figure 4, the total power consumption curve does not demonstrate a stable periodicity over time, and the peaks of power consumption differ greatly; the streak of the spike record is not clear from the right panel in Figure 4. Taken together, it can be speculated that the synchronization of the network is weak; however, this is an intuitive prediction based on the figure and cannot estimate the synchronization of the network quantitatively. Therefore, the following two synchronization indexes (the mean-max correlation coefficient ρ_*mean*_ and negative energy ratio α) are used for an enhanced description of the response of the network under the continuous stimulus.

**Figure 5:**
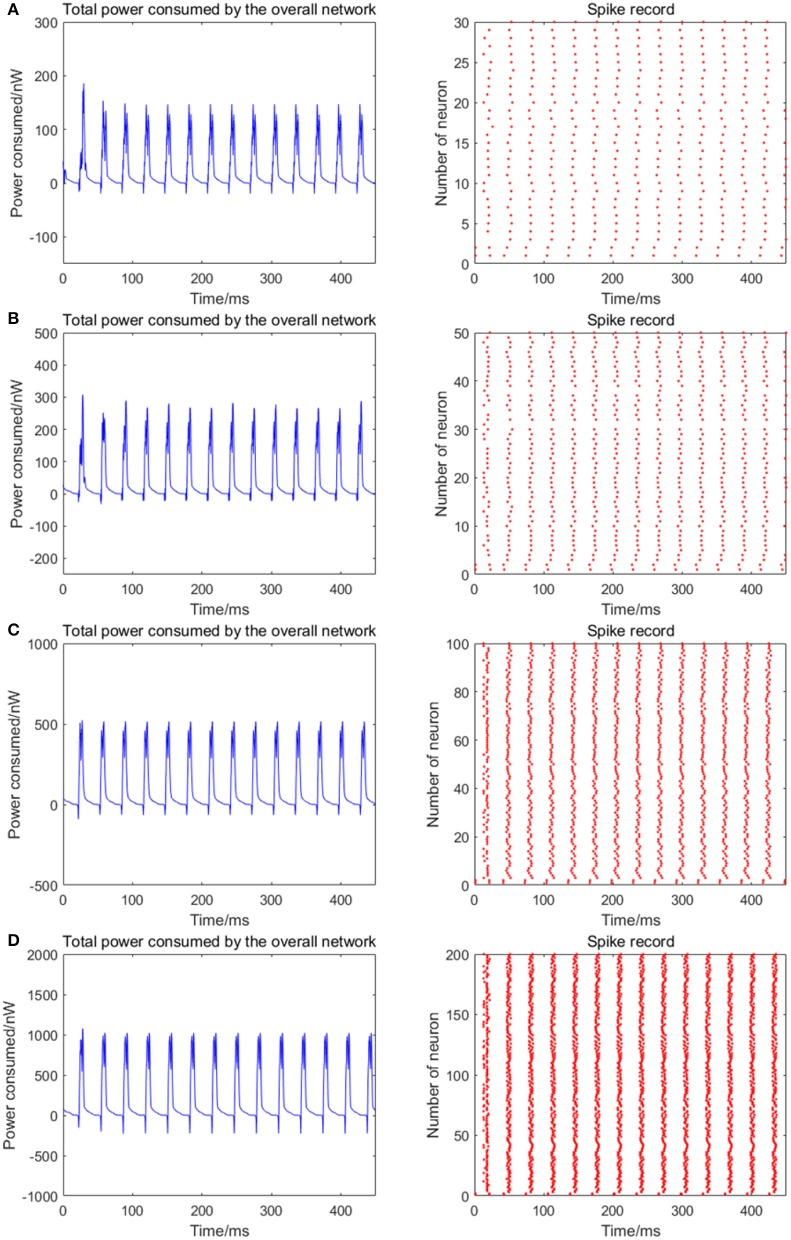
The total power consumption and the spike record of the overall network under continuous stimulus. The number of neurons in **(A–D)** are 30, 50, 100, 200, respectively. The coupling strength is uniformly distributed in [0, 0.5]; the signal transmitting delay is uniformly distributed in [0.3 ms, 1.8 ms]. The periodicity of the total power consumption curve is increasingly apparent and the spike record exhibits clear streaks with the increasing size of the network which indicates the synchronization of neuronal activity is getting stronger.

The number of neurons is increased to 30, 50, 100, and 200 in the network, where the 1st and 2nd neurons are continuously stimulated with the intensity of 10 μA/cm^2^ from *t* = 0–450 ms. The total energy consumption and the spike record of the overall network under continuous stimulus are shown in Figure 5. The corresponding negative energy ratio and MCC for Figure 5 is shown in Table 2.

**Table 2:** The corresponding negative energy ratio and MCC for Figure 5.

**Number of neurons**	**α (%)**	***ρ_*mean*_***
30	1.0453	0.8769
50	1.1452	0.9012
100	1.6556	0.9724
200	2.2379	0.9822

*I_ext_ = 10 μA/cm^2^. The coupling strength is uniformly distributed in [0, 0.5]; the signal transmitting delay is uniformly distributed in [0.3 ms, 1.8 ms]*.

According to Figure 5, when the distribution interval of the coupling strength and signal transmitting delay between neurons remains [0, 0.5] and [0.3 ms, 1.8 ms] unchanged, the periodicity of the total power consumption curve is increasingly apparent and the spike record exhibits clear streaks with the increasing size of the network. This indicates that the synchronization of neuronal activity is getting stronger. Based on the statistical results in Table 2, with the increase of the number of neurons, the corresponding MCC and negative energy ratio are both increasing monotonically, which shows that the raised synchronization of the network is in agreement with that in Figure 5. Combining Figure 5 and Table 2, it can be concluded that the distribution feature of the power consumption curve is closely related to the size of the network. That is, in the mutually coupling neural network under the continuous stimulus, the synchronization of the network and the periodicity of its power consumption curve are positively correlated to the number of neurons. Physiologically, the functional realization of the neural network depends on the common activity of a large number of neurons, and the neural population with the same function generally exhibits a high synchronization, thus the corresponding energy consumption changes periodically.

#### Correlation between energy distribution and coupling strength

**Figure 6:**
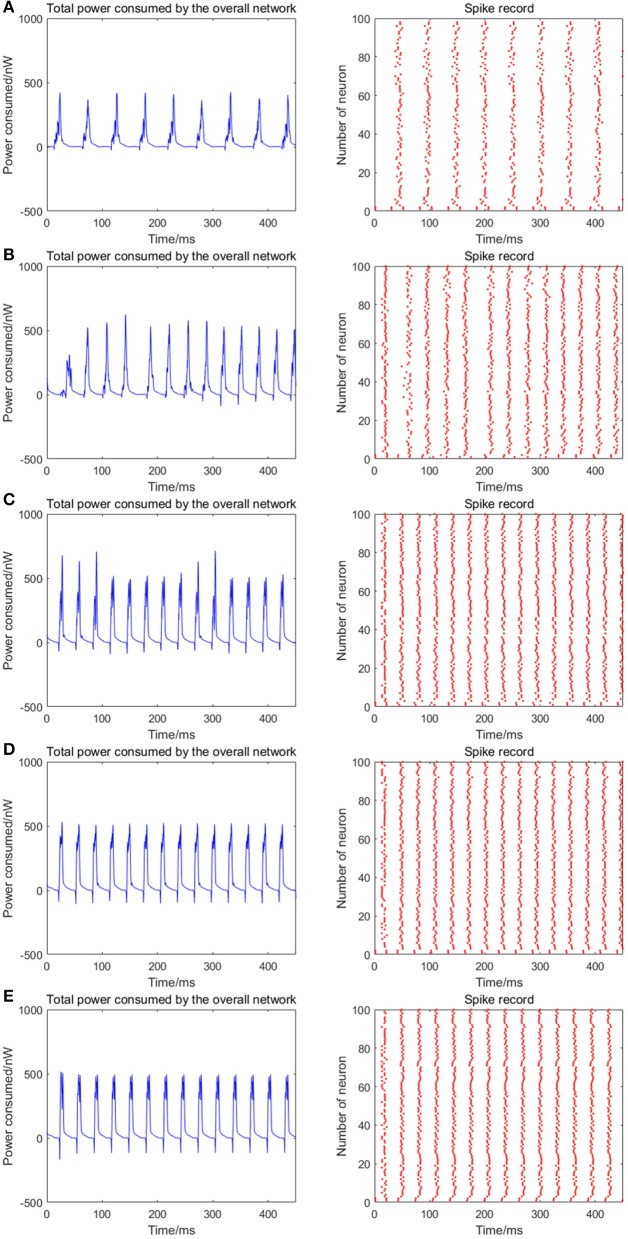
The total power consumption and the spike record of the overall network under continuous stimulus. The distribution interval of the coupling strength in **(A–E)** are [0, 0.05], [0, 0.1], [0, 0.3], [0, 0.5], [0, 1], respectively. The number of neurons is 100 and the signal transmitting delay is uniformly distributed in [0.3 ms, 1.8 ms]. The periodicity of the total power consumption curve and the synchronization of the network are positively correlated to the coupling strength.

Given the fully connected neural network consisting of 100 neurons, the signal transmitting delay between neurons is uniformly distributed in [0.3 ms, 1.8 ms]. The 1st and 2nd neurons are continuously stimulated with the intensity of 10 μA/cm^2^ from *t* = 0–450 ms. The total energy consumed by the overall network and the spike record under continuous stimulus is shown in Figures 6A–E when the coupling strength between the neurons is uniformly distributed in [0, 0.05], [0, 0.1], [0, 0.3], [0, 0.5], [0, 1], respectively. The corresponding negative energy ratio and MCC for Figure 6 is shown in Table 3.

**Table 3:** The corresponding negative energy ratio and MCC for Figure 6.

**Coupling strength**	**α (%)**	**ρ_mean_**
[0, 0.05]	0.4296	0.8292
[0, 0.1]	1.0215	0.7942
[0, 0.3]	1.5540	0.9381
[0, 0.5]	1.9046	0.9609
[0, 1]	2.2193	0.9692

*I_ext_ = 10 μA/cm^2^. The number of neurons is 100; the signal transmitting delay is uniformly distributed in [0.3 ms, 1.8 ms]*.

According to the results of Figure 6 and Table 3, as the coupling strength between the neurons increases, the periodicity of the total power consumption curve, MCC, negative energy ratio, and the synchronization reflected by spike record exhibit a consistent monotonicity. This indicates that the synchronous oscillation and energy distribution of the network are closely related to the coupling strength, that is, the synchronization of the network under identical conditions (the number of neurons and signal transmitting delay remains unchanged) and the periodicity of its power consumption curve are positively correlated to the coupling strength. It's consistent to the experimental result that the thalamocortical neurons with high-synaptic-strength exhibited well-synchronized activities during early sleep but exhibited a weak synchronization during late sleep due to the decreasing synaptic strength (Esser et al., 2007; Riedner et al., 2007). Since neural energy consumption reflects the law of global brain activity, the synchronous oscillation of neural populations corresponds to the periodic energy consumption of brain regions. Therefore, the larger the coupling strength, the more active the brain region and more salient the periodicity of energy consumption.

Notably, the negative energy ratio is extremely small, and the corresponding MCC is slightly large when the distribution interval of the coupling strength is [0, 0.05], which is inconsistent with the above conclusion. This might be attributed to the small coupling strength so that some neurons in the network exhibit subthreshold activities rather than firing spikes, while other neurons are the opposite that leads to a strong synchronization within their respective oscillation groups and a weak synchronization of the overall network. Thus, MCC which describes the synchronization within the oscillation groups is slightly large, while the negative energy ratio which describes the synchronization among the oscillation groups is extremely small. It's also clear in spike record from Figure 6 that the firing rate of neurons increases with the increase of coupling strength, which is manifested as the shortened oscillation period in the power consumption curve. This also shows that the neural energy can encode neural signals.

#### Correlation between energy distribution and signal transmitting delay

**Figure 7:**
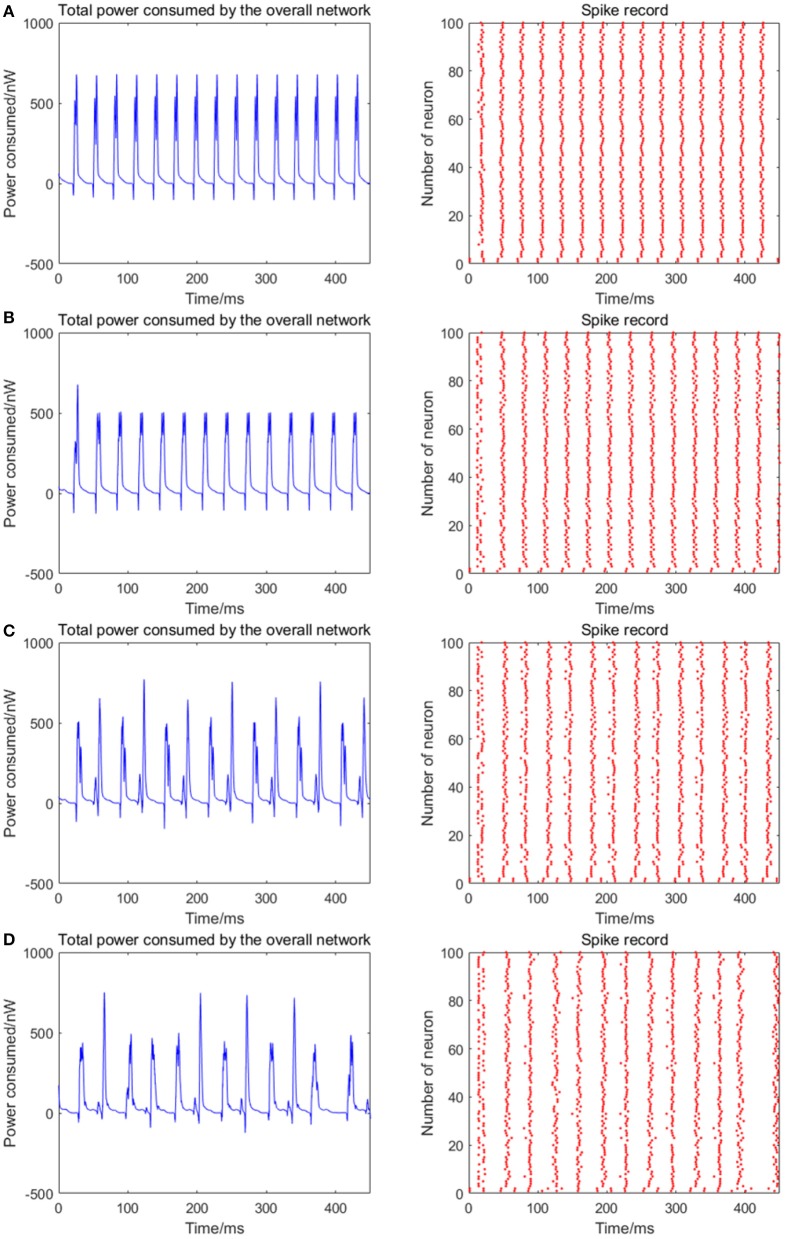
**(A)** The total power consumption and the spike record of the overall network under continuous stimulus. The distribution interval of the signal transmitting delay in **(A–D)** are [0.1 ms, 1.6 ms], [0.3 ms, 1.8 ms], [0.5 ms, 2.0 ms], [0.7 ms, 2.2 ms], respectively. The number of neurons is 100 and the coupling strength is uniformly distributed in [0, 1]. The periodicity of the total power consumption curve and the synchronization of the network are negatively correlated to the signal transmitting delay.

Given the fully connected neural network consisting of 100 neurons, the coupling strength between the neurons is uniformly distributed in [0, 1]. The 1st and 2nd neurons are continuously stimulated with the intensity of 10 μA/cm^2^ from *t* = 0–450 ms. The total energy consumed by the overall network and the spike record under continuous stimulus is shown in Figures 7A–D when the signal transmitting delay between neurons is uniformly distributed in [0.1 ms, 1.6 ms], [0.3 ms, 1.8 ms], [0.5 ms, 2.0 ms], [0.7 ms, 2.2 ms], respectively. The corresponding negative energy ratio and MCC for Figure 7 is shown in Table 4.

**Table 4:** The corresponding negative energy ratio and MCC for Figure 7.

**Signal transmitting delay (ms)**	**α (%)**	**ρ_*mean*_**
[0.1, 1.6]	2.2244	1.3674
[0.3, 1.8]	1.9132	0.9648
[0.5, 2.0]	1.8743	0.8989
[0.7, 2.2]	1.3674	0.7498

*I_ext_ = 10 μA/cm^2^, the number of neurons is 100; the coupling strength is uniformly distributed in [0, 1]*.

According to the results of Figure 7 and Table 4, as the signal transmitting delay between neurons approaches 0, the periodicity of the total power consumption curve becomes salient. In addition, MCC and negative energy ratio increase simultaneously, and the spike record exhibits a streak-like feature. This indicates the close relationship between signal transmitting delay and synchronous oscillation with the energy distribution of the network, that is, the synchronization of the network under identical conditions (the number of neurons and coupling strength remains unaltered) and the periodicity of its power consumption curve are negatively correlated to the signal transmitting delay. Since the signal transmitting delay represents the lag from the release of excitatory neurotransmitters by the presynaptic to the postsynaptic neurons receiving neurotransmitters, in the case of prolonged lag, the correlation of neuronal activities is weak, thereby affecting the synchronization activity of the overall network and the periodicity of energy consumption. The change in signal transmitting delay can also affect firing rate of the network that the shorter the signal transmitting delay, the higher the firing rate.

### The relationship between network parameters and neural energy

#### The relationship between the number of neurons and neural energy

**Figure 8:**
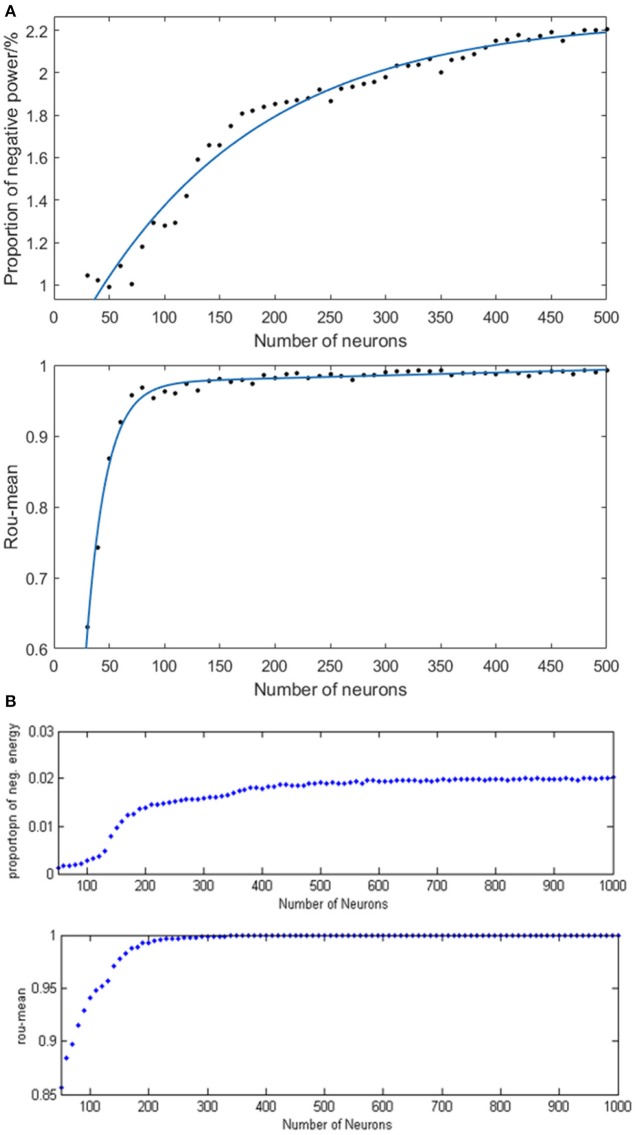
**(A)** The curve of α and ρ_*mean*_ varies as a function of the number of neurons in the network based on the Hodgkin–Huxley model; **(B)** The curves of α and ρ_*mean*_ vary as a function of the number of neurons in the network based on Wang–Zhang biophysics model (Wang et al., 2006; Wang and Wang, 2014).

In order to study the specific relationship between the number of neurons (30–500) and neural energy, we simulated the neural network where the coupling strength and the signal transform delay were distributed uniformly in [0, 0.5] and [0.3 ms, 1.8 ms], respectively. The corresponding negative energy ratio and MCC are shown in Figure 8A of which, each point is obtained as follows: for the number of neurons*N*, simulate any two neurons in the network with the intensity of 10 μA/cm^2^ from *t* = 0–450 ms and calculate the negative energy ratio α and MCCρ_*mean*_. This process is repeated 5 times for the average $\bar{\alpha }$ and $\bar{\rho_{mean}}$ as vertical coordinates corresponding to *N* neurons in Figure 8A. Figure 8B is the result of the structural neural network based on the Wang–Zhang biophysical model in the previous study.

According to Figure 8A, a monotonous relationship occurs between the number of neurons and the negative energy ratio with MCC that these two synchronization indexes are elevated with the increasing number of neurons, thereby indicating the increasingly synchronous oscillation of the network. This phenomenon suggests that the energy coding is also capable of representing the network activity synchronization and is highly consistent with the traditional measure of the correlation coefficient. Physiologically, the synchronous oscillation of the network consisting of a large number of neurons requires a high energy supply which means more energy storage, and the negative energy ratio reflects the stored energy in the network activity; hence, it can also reveal the state of network synchronization similar to the conventional measure of the correlation coefficient. However, the curve of MCC achieves saturation early, which is caused by the saturated synchronization of the network when the number of neurons is increased to 100, while the saturation for the curve of negative energy ratio occurs after *N* > 400. Therefore, the analysis of energy coding is superior to that of correlation coefficient. The slower tendency of saturation for negative energy ratio enables it to distinguish the number of neurons in the network effectively. The comparison with the result based on the Wang–Zhang biophysical model (Wang et al., 2006; Wang and Wang, 2014) shows that the network property displayed by both neuronal models is nearly consistent despite the difference in the computational values due to intrinsic differences between the two models. In addition, the simulated network based on the H–H model consists maximally 500 neurons due to computational complexity.

#### The relationship between coupling strength and neural energy

**Figure 9:**
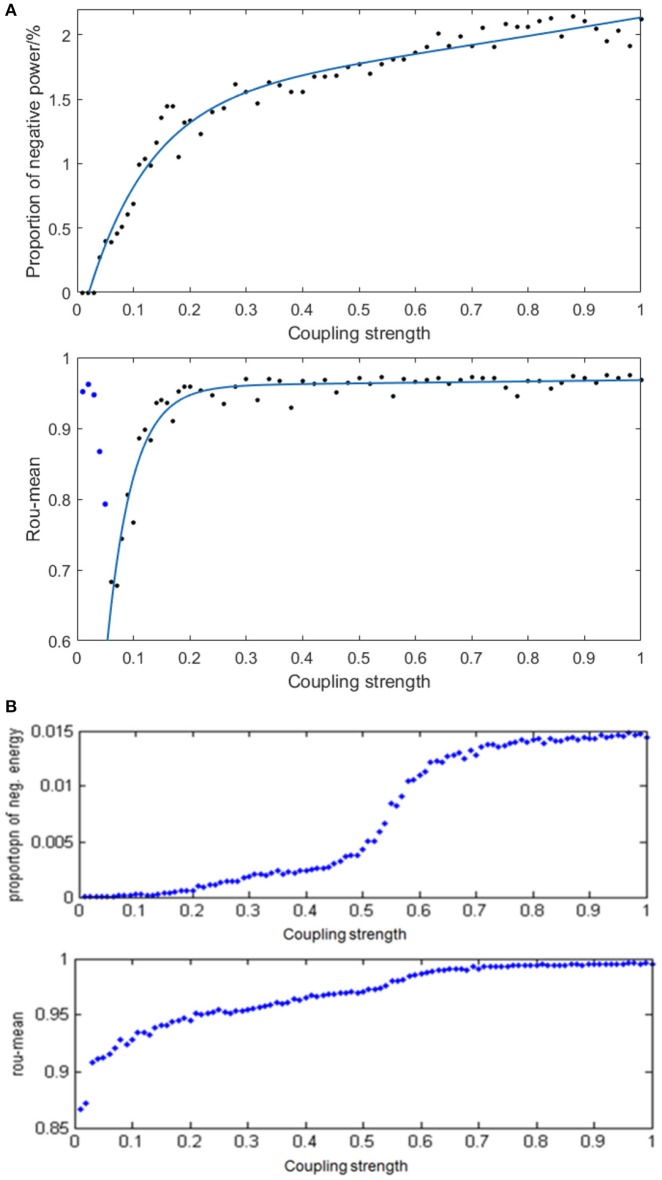
**(A)** The curve of α and ρ_*mean*_ varies with different distribution intervals of coupling strength in the network based on the Hodgkin–Huxley model; **(B)** The curves of α and ρ_*mean*_ varies with different distribution intervals of coupling strength in the network based on Wang–Zhang biophysical model (Wang et al., 2006; Wang and Wang, 2014).

Given the fully connected neural network consisting of 100 neurons and the signal transmitting delay uniformly distributed in [0.3 ms, 1.8 ms], we simulated the neural network with different distribution intervals of coupling strength and calculated the corresponding negative energy ratio as well as MCC. Figure 9A demonstrates the specific relationship between the coupling strength and neural energy. Each point in Figure 9A is obtained as follows: for each distribution interval of the coupling strength [0,*x*] (*x* take totally 60 values as 0.01, 0.02,…, 0.20, 0.22, … 1 that represents the horizontal coordinate of corresponding point), any two neurons in the network are stimulated with the intensity of 10 μA/cm^2^ from *t* = 0–450 ms and the negative energy ratio α and MCC ρ_*mean*_ were calculated. The process is repeated 5 times and the average $\bar{\alpha }$ and $\bar{\rho_{mean}}$ serve as vertical coordinates in Figure 9A. Figure 9B is the result of the structural neural network based on Wang–Zhang biophysical model in the previous study.

According to Figure 9A, when *x* > 0.05, both the negative energy ratio and MCC monotonically increase with the increasing distribution interval of coupling strength, which indicates the increasing synchronization of the network. In addition, both curves exhibit a gradual saturation as the coupling strength increases; however, the curve of the negative energy ratio reaches saturation slowly relative to the curve of MCC. When *x* ≤ 0.05, the negative energy ratio is small, while MCC is abnormally large, which is not in agreement with the above analysis. This phenomenon might be attributed to the small coupling strength between neurons, which results in the subthreshold state of most of the neurons instead of firing spikes. And other neurons are the opposite, which leads to a strong synchronization within their respective oscillation groups but a weak synchronization of the overall network. Thus, it manifested as that the negative energy ratio which describes the synchronization among oscillation groups is small, while the MCC which describes the synchronization within oscillation groups is large. This is also one of the advantages of energy coding. Moreover, the comparison with the result based on Wang–Zhang biophysical model (Figure 9B) shows a nearly consistent dynamic behavior of the network. Since the coupling strength between neurons affects their information interaction and this process relies on energy consumption for completion, it can be speculated that the stronger the coupling strength, the more synchronous the network activity and higher the demand for energy which means more energy storage that can be described by the negative energy ratio. Thus, the negative energy ratio can be regarded as a measure of network synchronization.

#### The relationship between signal transmitting delay and neural energy

**Figure 10:**
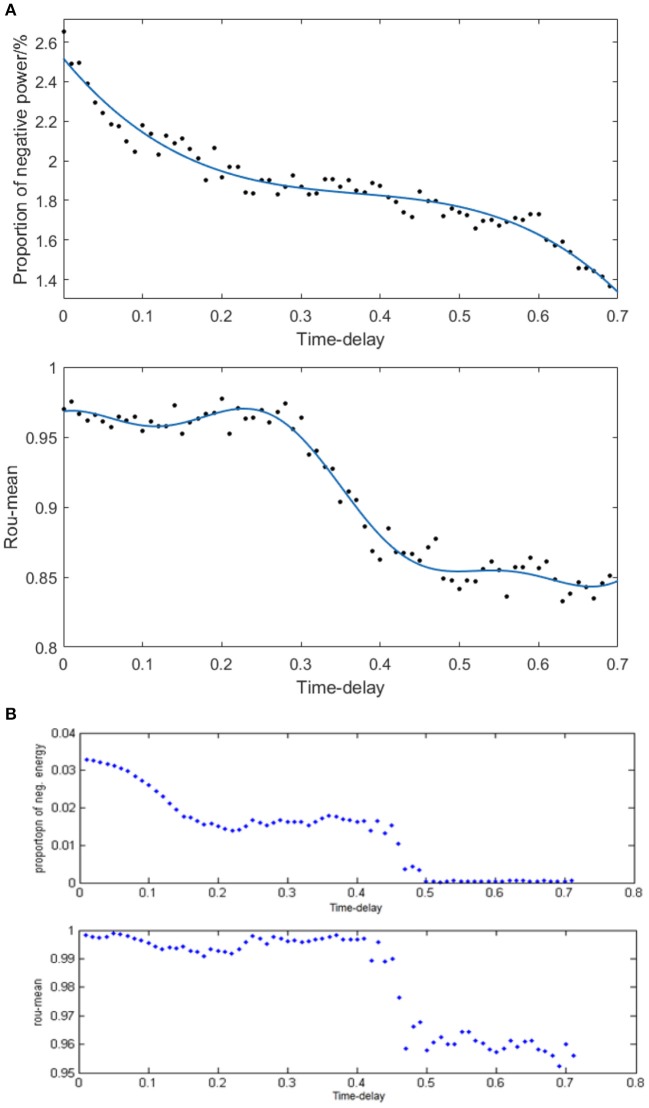
**(A)** The curve of α and ρ_*mean*_ varies with different distribution intervals of signal transmitting delay in the network based on the Hodgkin–Huxley model; **(B)** The curve of α and ρ_*mean*_ varies with different distribution intervals of signal transmitting delay in the network based on Wang–Zhang biophysical model (Wang et al., 2006; Wang and Wang, 2014).

Given the fully connected neural network consisting of 100 neurons and the coupling strength uniformly distributed in [0, 1], we simulated the neural network with various distribution intervals of signal transmitting delay and calculated the corresponding negative energy ratio as well as MCC shown in Figure 10A to study the specific relationship between signal transmitting delay and neural energy. Each point in Figure 10A is obtained as follows: for each distribution interval of signal transmitting delay [*x*,*x* +1.5] ms (*x* takes 70 different values as 0, 0.01, 0.02,… 0.69 that represents the horizontal coordinate of the corresponding point), any two neurons in the network are simulated with the intensity of 10 μA/cm^2^ from *t* = 0–450 ms and the negative energy ratio α and MCC ρ_*mean*_ are calculated. This process is repeated 5 times to obtain the average $\bar{\alpha }$ and $\bar{\rho_{mean}}$ as vertical coordinates in Figure 10A. Figure 10B is the result of the structural neural network based on Wang–Zhang biophysical model in the previous study.

According to Figure 10A, both the negative energy ratio and MCC decrease as the distribution interval of signal transmitting delay increases which means the weakening synchronization of the network. This simulation result can be attributed to the prolonged lag from the release of excitatory neurotransmitters by the presynaptic neuron to the postsynaptic neuron receiving neurotransmitters which decreases the activity correlation between the presynaptic neuron and the postsynaptic neuron. This phenomenon results in less salient synchronization activity of the overall network that requires less energy supply; thus, the stored energy in the network activity is less, and the corresponding negative energy ratio is low. More specifically, both the curves of the negative energy ratio and MCC can be roughly divided into three stages. In the first stage corresponding to the horizontal coordinate at [0, 0.3], the curve of negative energy ratio declines continually as the distribution interval of signal transmitting delay moves away from zero; however, the curve of MCC is maintained at a stable horizontal state. This could be ascribed to the synchronization among oscillation groups in the network that weakens gradually with the increase in signal transmitting delay, while the synchronization within each oscillation group maintains a high level within this interval. In the second stage corresponding to the horizontal coordinate at [0.3, 0.5], the curve of negative energy ratio shows no apparent tendency to decline, whereas the curve of MCC declines rapidly as the signal transmitting delay increases. This phenomenon indicates that the increase in signal transmitting delay greatly reduces the synchronization within each oscillation group but has almost no influence on the synchronization of the overall network. And the situation in the third stage corresponding to the horizontal coordinate at [0.5, 0.7] is similar to that of the first stage. These features are consistent with the results based on Wang–Zhang biophysical model (Figure 10B) that the negative ratio and MCC of the network alter in stages with the variation of the signal transmitting delay. Therefore, there exists a close relation between the signal transmitting delay and the oscillation groups in the network, and combining the analysis of energy coding and correlation coefficient can improve the understanding of the operations of the network.

## Conclusions

The cognitive neural structure of the brain is complex and multi-hierarchical; thus, it is essential to combine various scales and hierarchies for investigating the neural coding of the cerebral cortex (Abbott, 2008). Especially, the effective theory of neural coding should be proposed from the global view of the brain activity. The neural energy coding provides an excellent solution for combining different hierarchies and establishing a global model of brain function (Wang R. et al., 2014; Wang and Zhu, 2016).

The current study investigated the action potential of a single neuron and the synchronous oscillation of a structural neural network by neural energy applying the H–H neuronal model. We obtained the preliminary conclusions about the energy consumption of an action potential and the quantitative relationship among synchronous oscillation, energy consumption, and network parameters (number of neurons, coupling strength, and signal transmitting delay) as follows:

1. In the course of firing an action potential, the neuron firstly stores energy before the peak of the action potential and then consumes energy. And in the power consumption curve, the negative energy, which means the energy stored by the neuron from ATP hydrolysis, makes up only a small proportion of the total energy consumed by the neuron. This neuronal work mechanism can explain the physiological phenomenon that the blood flow rises by about 31% while the accompanying oxygen consumption increases by only 6% in the stimulation-induced neural activity, which is consistent with the previous research findings (Wang R. et al., 2014).
2. The synchronization of the network and the periodicity of the network energy distribution is positively correlated to the number of neurons and coupling strength, but negatively correlated to signal transmitting delay. In fact, well-synchronized network activities resulting from the change of these network parameters will lead to similar power consumption curves of each neuron in course of time which constitute periodic energy consumption of the network.
3. The proportion of negative energy in power consumption curve was positively correlated to the synchronous oscillation of the neural network. From the biological point of view, the stronger synchronous oscillation of the neural network demands for excessive energy supplies which means more energy storage, and the negative energy ratio reflects the stored energy in the network activity. Therefore, the energy index of negative energy ratio can be used to describe the dynamic properties of the network, and the energy coding has great superiorities for exploring the operation of the network further.
4. In addition, we compared the simulation result of the structural neural network based on H–H model with the counterpart based on Wang–Zhang biophysical model (Wang et al., 2006; Wang and Wang, 2014) and found almost identical dynamic properties and energy coding characteristics of the network in both models. This suggests that the H–H model is essentially similar to the Wang–Zhang biophysical model despite different levels at which both are constructed. The former is constructed at the level of the molecule, while the latter is directly established at the level of neurons. Considering the computational complexity, the result based on the H–H model requires more time as compared to the Wang–Zhang biophysical model, which is the limitation of the H–H model. On the other hand, the advantage of the H–H model is that it can obtain precise calculation results, whereas that of the Wang–Zhang biophysics model is that it can obtain the membrane potential function as well as energy function of neurons (Wang et al., 2006; Wang R. et al., 2014; Wang and Zhu, 2016); the H–H model can obtain only the numerical solution.

In the field of experimental neuroscience, a technical record of the membrane potential of each neuron anatomically in order to study the cortical network comprising of a plethora of neurons is challenging and cost-ineffective (Hipp et al., 2011). However, it's possible to calculate the power consumption of hundreds of neurons in some functional region by the electrophysiological records of the membrane potential of a small number of neurons. Due to the scalar property of energy, the energy consumption of local cortical network can be estimated to study the formation mechanism of the cognitive functional network cost-effectively according to the density of neurons (Wang et al., 2006, 2008, 2016, 2017; Wang and Wang, 2014; Wang R. et al., 2014; Wang Z. et al., 2014; Yan et al., 2016; Zheng et al., 2016). Taken together the neural theory of energy coding has great potential and can significantly influence the study of encoding and decoding in cognitive neuroscience.

## Author contributions

Modeling and simulation: ZZ; Result analysis and discussion: ZZ, RW, and FZ; Writing and modification of the paper: ZZ and RW. All authors approved it for publication.

### Conflict of interest statement

The authors declare that the research was conducted in the absence of any commercial or financial relationships that could be construed as a potential conflict of interest.
